# Serious Shortcomings in the Management of Children with Anaphylaxis in Scottish Schools

**DOI:** 10.1371/journal.pmed.0030326

**Published:** 2006-08-22

**Authors:** Kirsty E Rankin, Aziz Sheikh

**Affiliations:** Division of Community Health Sciences, General Practice Section, University of Edinburgh, Edinburgh, United Kingdom; Wythenshawe Hospital, United Kingdom

## Abstract

**Background:**

The United Kingdom incidence of anaphylaxis has increased very sharply over the last decade, with the highest rates of hospital admissions occurring in school-aged children. This raises concerns about the extent to which schools are aware of approaches to the prevention and treatment of anaphylaxis.

****Methods and Findings**:**

We undertook a national postal survey of 250 Scottish schools enquiring about approaches to managing children considered to be at risk of anaphylaxis. We obtained responses from 148 (60%) schools, 90 (61%) of which reported having at least one at risk child. Most (80%) schools with children considered to be at risk reported having personalised care plans and invariably reported having at least one member of staff trained in the emergency treatment of anaphylaxis. Access to adrenaline was available on-site in 97% of these schools. However, significantly fewer schools without children considered to be at risk reported having a trained member of staff (48%, *p <* 0.001), with access to adrenaline being very poor (12%, *p <* 0.001). Overall, 59% of respondents did not feel confident in their school's ability to respond in an emergency situation.

****Conclusions**:**

Most schools with children considered to be at risk of anaphylaxis report using personal care plans and having a member of staff trained in the use of, and with access to, adrenaline. The picture is, however, less encouraging in schools without known at risk children, both in relation to staff training and access to adrenaline. The majority of schools with at risk children have poorly developed strategies for preventing food-triggered anaphylaxis reactions. There is a need for detailed national guidelines for all schools, which the Scottish Executive must now ensure are developed and implemented.

## Abstract 2

Schools whose officials know that some of their pupils have serious allergies are often prepared to deal with emergencies, but many don't have good plans for exposure prevention. When there is no knowledge of specific pupils at risk, neither preparedness nor prevention efforts are widespread.

## Editors' Summary

**Background.**

Anaphylaxis is a severe allergic reaction that can cause a drop in blood pressure and swelling of the body tissues (swelling of the neck and throat and narrowing of the airways can make it hard to breathe). The reaction can be triggered by foods, such as peanuts or eggs, or by bee stings, natural latex (rubber), and certain drugs such as penicillin. Foods are the most common trigger in children. In the United Kingdom, thousands of people develop anaphylaxis each year, and the number appears to be increasing. If anaphylaxis is severe, a patient will need to be hospitalized, and the highest risk of hospitalization is in school-aged children. About 20 people die each year in the United Kingdom from anaphylaxis. Anaphylaxis can develop both in people known to have a tendency (risk) of getting the condition and in those with no known risk.

**Why Was This Study Done?**

The researchers wanted to find out how many school children there were in Scotland with a known risk of developing anaphylaxis. They also wanted to know how many schools were taking steps to minimize these children's risk of getting anaphylaxis during school hours (the risk can be reduced, for example, by prohibiting children from sharing food and eating utensils). Finally, they wanted to find out how many schools had the necessary equipment and trained staff to treat a child that develops anaphylaxis on site.

**What Did the Researchers Do and Find?**

The researchers sent a questionnaire in the mail to 250 Scottish schools. They received replies from 148 schools, of which 90 reported having at least one child with a known risk of developing anaphylaxis. Most of the schools (80%) that had children at risk had at least one staff member trained to treat anaphylaxis, and 97% of these schools had the drug adrenaline (epinephrine) on site, which is a lifesaving medication for treating anaphylaxis. But many of these schools were doing poorly at reducing the risk of anaphylaxis—for example, only one in three of these schools banned the sharing of eating utensils. Another worrying finding was that among the 58 schools that did not have children known to be at risk of anaphylaxis, less than half had a trained member of staff, and only about one in eight of these schools had adrenaline on site.

**What Do These Findings Mean?**

It is reassuring that most schools in Scotland that have children known to be at risk of anaphylaxis are well equipped to deal with this emergency if it occurs. But many of these schools are not doing enough to reduce the risk of anaphylaxis occurring. It is worrying that schools that don't have children known to be at risk are often very poorly equipped to deal with anaphylaxis. One weakness of this study is that 102 schools never replied to the questionnaire, so we can't know how well prepared these schools are for dealing with anaphylaxis. Nevertheless, the study suggests that there needs to be detailed guidance for all schools in Scotland on preventing and treating anaphylaxis.

**Additional Information.**

Please access these Web sites via the online version of this summary at http://dx.doi.org/10.1371/journal.pmed.0030326.

- NHS Direct page on anaphylaxis
- MedlinePlus page on anaphylaxis
- Mayo Clinic page on anaphylaxis
- Wikipedia entry on anaphylaxis  (note: Wikipedia is a free Internet encyclopedia that anyone can edit)

## Introduction

Anaphylaxis is an acute, potentially life-threatening allergic reaction due to a systemic allergic reaction [1]. Thousands of people develop anaphylaxis each year in the United Kingdom, and of these about 20 die annually [2–5]; the majority of these reactions and deaths are believed to be preventable.

Historically, the condition has been defined in mechanistic terms as a hypersensitivity reaction involving release of inflammatory mediators from mast cells and basophils following allergen interaction with cell-bound immunoglobulin E ; in contrast, anaphylactoid reactions have been described as involving non-immunoglobulin E–mediated release of inflammatory mediators [6]. It is widely recognised that the clinical picture and emergency treatment of anaphylaxis are similar regardless of patho-physiological mechanism [7].

There is currently renewed interest in developing and standardising a clinical definition of anaphylaxis, and important progress has been made in recent years [8,9]. Cutaneous, gastrointestinal, and neurological symptoms and signs are recognised as part of the clinical spectrum [10]; however, it is cardiovascular and respiratory symptoms that are the most important, as these are most frequently implicated in fatal reactions [5].

Reliable data on the epidemiology of anaphylaxis in the general population are difficult to obtain [2–4,11–16], but it appears that the UK incidence of anaphylaxis has increased very markedly over the last decade [2,3]. A number of triggers are recognised, these including food, drugs, venom, latex, and exercise. These vary considerably with age, with foods being the most common trigger in children [17].

In view of the substantial numbers of children now at risk of anaphylaxis and the risks of being exposed to food and other triggers in the school setting, there has been international interest in developing guidelines and approaches for the prevention and treatment of anaphylaxis. There is, however, very little known about the preparedness of UK schools to respond to anaphylaxis should it develop and also, importantly, what approaches schools are taking to minimise the risks of these reactions being triggered. Such information is important in its own right, but is also timely, as allergy services are being reviewed by the Scottish Executive and Department of Health in the aftermath of the recent damning report from the Parliamentary Health Select Committee, which concluded that “serious problems exist in the current provision of allergy services” [18].

In order to investigate these issues we undertook a survey of Scottish schools enquiring about the numbers of children considered to be at risk of anaphylaxis, the schools' approaches to minimising the risk of food-triggered allergic reactions, and emergency treatment provisions if reactions do occur.

## **Methods**

### Study Design

We sent a self-completed semi-structured postal questionnaire to Scottish schools in August–September 2005.

### Questionnaire Design and Piloting

The questionnaire was based on current models of “best practice” for the treatment and prevention of anaphylaxis in situ around the world [19–22]. We piloted the questionnaire amongst teachers from different schools, and the feedback obtained helped in further refining the instrument (see Text S1 for final questionnaire).

### Sampling Strategy

The Scottish Executive's database of all 2,855 schools in Scotland was used to select a sample of 250 Scottish schools. This database geographically and administratively divides schools into 32 Local Education Authorities. Stratified random sampling was employed in order to ensure that we sampled from all Local Educational Authorities, and as comparable numbers of schools were in each strata, we then used computer-generated random sampling to sample ~9% of schools within each strata.

Permission to carry out the survey was sought and obtained from each Local Educational Authority. In the majority of cases the decision to partake was left to the discretion of the individual school. Selected schools were sent a copy of the cover letter, confidential questionnaire, stamped addressed envelope, and also an option to fax back responses. The letter was marked for the attention of head teachers, inviting them to complete it or forward it on to the most appropriate person within the school.

Non-responders to this initial mailing (*n =* 134) were followed up with an E-mail enquiring whether or not they had received the questionnaire and if not they were sent another copy. Those who still failed to respond (*n =* 124) were mailed a second and final invitation letter and another copy of the questionnaire. We telephoned a 10% (*n =* 10) random sample of the 100 schools that failed to respond to any of our invitations in an attempt to ascertain reasons for their failing to respond.

### National Data on Scottish Schools

In order to assess the representativeness of responding schools, we obtained national databases on types of school (primary, secondary, or special—this last category indicating schools responsible for the education of children with behavioural problems and/or learning difficulties), gender of pupils taught (single sex or co-educational), funding source (state or independent), and geographical location (urban or rural, measured using six categories: remote town, remote rural, other urban, large urban, accessible town, and accessible rural) from the Scottish Executive's Schools Division. School postcode was used to assign each school a deprivation category (DEPCAT) using the Carstairs index [23]. This is a composite measure of four variables derived from 2001 census small-area statistics: the extent of overcrowding, male unemployment, low social class, and having no car. Seven DEPCAT groups are defined, these ranging from one (the most affluent) to seven (the most deprived) [24].

### Analysis

Descriptive statistics were used to summarise results, with the chi-square test used for comparisons of categorical data. For free text responses, we used the principles of qualitative content analysis to develop a coding frame and identified key emerging themes [25].

## Results

Two of the schools in our sample were no longer operational, bringing the available sample size down to 248. We obtained responses from 60% (*n =* 148) of schools, the majority of which were completed either by head teachers or deputy head teachers. At least one school responded from each Local Educational Authority. Enrolment figures from the Scottish Executive database revealed that responding schools had a total of 65,185 registered pupils.

The key overall characteristics of Scottish schools are detailed in Table 1. Table 2 compares the characteristics of responding schools with those of all schools in Scotland on a broad range of parameters and shows that responding schools were representative of schools nationally with respect to the type of school, gender of pupils taught, funding mechanism, geographical location, and socio-economic status. The main reasons cited by schools for non-response were “lack of time” and “receiving too many questionnaires”.

**Table 1:**
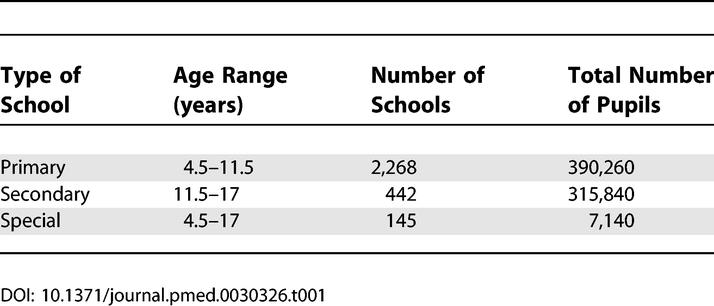
Characteristics of Scottish Schools (2005)

**Table 2:**
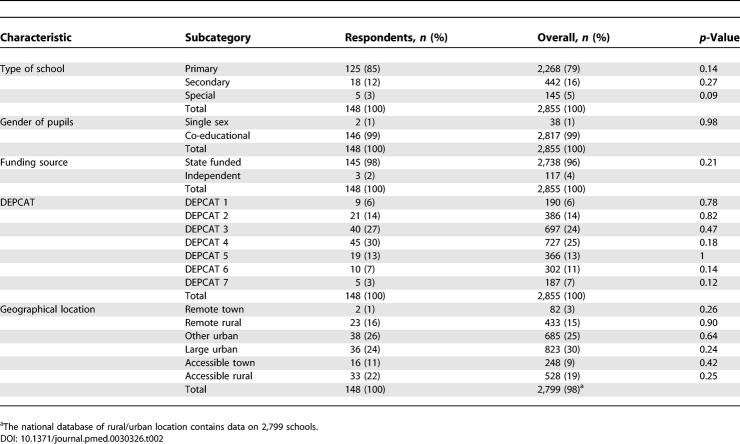
Comparison of Characteristics of Responding Schools with Scottish Schools Overall

Ninety (61%, 95% confidence interval [CI] 53–68) schools reported having one or more child known (on the basis of previous anaphylaxis and/or prescription of self/carer-administered adrenaline) to be at risk of experiencing anaphylaxis. Schools were asked to detail the absolute number of pupils considered to be at risk; those that indicated that they had at risk pupils but failed to give an absolute number were assumed to have one such pupil enrolled. A minimum of 282 pupils were known to schools as having a history of anaphylaxis or being prescribed an adrenaline auto-injector, giving a minimum prevalence estimate of school-aged children considered to be at risk of anaphylaxis of 0.4% (95% CI 0.3–0.5).

Most (80%, 95% CI 71–87) of the 80 schools with children considered to be at risk reported having personalised care plans in place, and these schools invariably (100%) had one or more staff members who had received training in the emergency management of anaphylaxis. Access to adrenaline was available on-site in 97% (95% CI 91–99) of these schools. However, significantly fewer schools without children considered to be at risk reported having a trained member of staff (48%, 95% CI 38–58 *p <* 0.001), and access to adrenaline was very poor in these schools (12%, 95% CI 6–23; *p <* 0.001). Overall, 59% (95% CI 51–66) of respondents did not feel fully confident in their school's ability effectively to respond in an emergency situation.

There was considerable scope for improvement in preventative strategies to minimise the risk of triggering allergic reactions in schools with children considered to be at risk (Table 3).

**Table 3:**
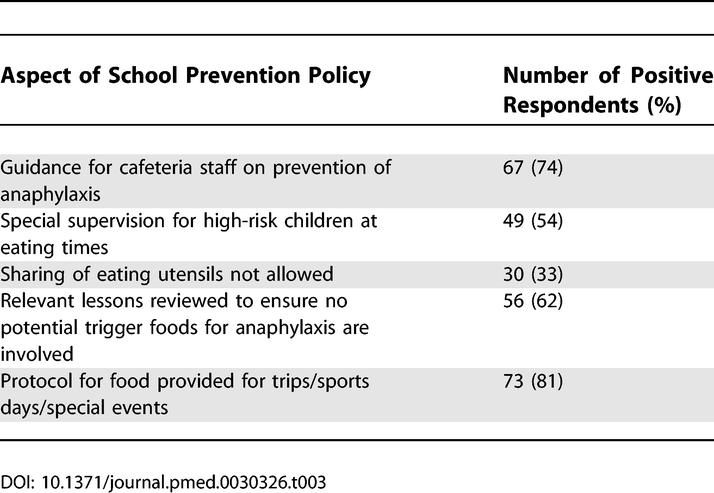
Strategies for the Prevention of Anaphylaxis Reactions in Schools with Children Considered to Be at Risk of Anaphylaxis (*n =* 90)

Overall, the majority of schools (78%, 95% CI 70–84) expressed a need for detailed national guidelines on appropriate standards of care for the prevention and emergency management of anaphylaxis in schools.

Analysis of free text responses revealed that particular concerns centred around the appropriateness of instituting “no nut” policies, the practicalities of alerting all members of staff to at risk children, implementing “no food sharing” policies, ensuring ready access to adrenaline throughout the school environment, and access to and funds for training (and periodic re-training) of staff. Some schools with no children considered to be at risk expressed concern that, even if trained, staff would not have access to adrenaline if a child unexpectedly developed anaphylaxis.

## Discussion

### Main Findings

The majority of Scottish schools now have at least one child considered to be at risk of developing anaphylaxis within the school setting, and although most of these schools have trained staff with access to emergency medication and personalised care plans for these children, they continue to express concern about their ability to respond effectively in an emergency situation. The picture is even more concerning in schools that currently do not know of at risk children, because of possible under-recognition and the risk that children may develop their first reaction whilst at school. Typically, there will be no access to adrenaline in such cases.

### Relevant Contextual Considerations

Most children at risk of anaphylaxis in the UK are managed by their general practitioners, and many are prescribed an automatic adrenaline preparation for use in an emergency. Adrenaline is only available on prescription, and the most commonly prescribed preparation in the UK is the EpiPen auto-injector. Schools do not have access to generic supplies of adrenaline and will therefore generally only have access to adrenaline if parents make this available for use for individual children. National guidance from the Scottish Executive advises that Local Educational Authorities encourage the use of personal care plans developed in conjunction with health-care professionals, schools, parents, and, where possible, pupils. This guidance also advises that Local Educational Authorities provide insurance cover to suitably trained staff who volunteer to administer medication, but makes clear that “school staff should ***never*** administer medication without appropriate training from health professionals” [26]. The legal status of this guidance is, however, unclear.

### Strengths and Limitations of This Work

The strengths of this study include its national sampling frame and its focus on issues relating to both the prevention and emergency management of anaphylaxis; previous UK and US studies have had narrower and more regional foci [27,28]. The study's main limitation is the sub-optimal response rate—however, our comparison of the characteristics of responding schools with those of all Scottish schools shows that our sample was representative of schools across Scotland. Furthermore, our investigation of a sample of non-responders suggests that non-response largely reflects a lack of interest in the subject under study, which, although of concern, is unlikely to have altered the main conclusion of overall deficiencies in schools' preparedness to prevent and manage anaphylaxis in children. It is also important to note that the estimate of the prevalence of children considered to be at risk of anaphylaxis is likely to represent an under-estimate of the true prevalence of high-risk children as our data refer only to children known to schools to be at risk, and it is for this reason that we describe this as a minimum estimate of prevalence.

### Conclusions

The majority of responding schools articulated a need for detailed advice on the management of children with anaphylaxis, which the Scottish Executive must now ensure is developed. This advice should cover food allergen avoidance policies, early recognition of anaphylaxis, and crisis management [29]. More specifically, personalised care plans for all children considered to be at risk and at least two members of staff trained in the recognition of and emergency treatment of anaphylaxis with ready access to adrenaline in every Scottish school are, we suggest, minimum standards of care. Crucially, the resources must also be made available to schools to implement any necessary policy changes, and for Local Educational Authorities to ensure that these standards are met, for this potentially fatal, but usually avoidable and eminently treatable condition.

## Supporting Information

Text S1 Questionnaire: Prevention and Treatment Protocols for Anaphylaxis in Scottish Schools (268 KB DOC)

## Abbreviations

CI: confidence interval
DEPCAT: deprivation category
