# Supplementary material for: Serious Shortcomings in the Management of Children with Anaphylaxis in Scottish Schools
Source: PLoS Med. 2006 Aug 22;3(8):e326. doi: 10.1371/journal.pmed.0030326 (PMC1551918; doi:10.1371/journal.pmed.0030326)
Supplement: Supplementary file 1 [file pmed.0030326.sd001.doc]

**Appendix 1: Questionnaire**

**PREVENTION AND TREATMENT PROTOCOLS FOR ANAPHYLAXIS IN SCOTTISH SCHOOLS**

**Anaphylaxis is a severe, life threatening allergic reaction which affects around 2% of the population and numbers are on the increase. It is a risk for those who suffer from allergies but can also occur in somebody who has perfect health. I am a fourth year medical student at the University of Edinburgh and I am interested in the provision of care for children suffering from this potentially life threatening condition. Anaphylaxis commonly occurs in childhood and currently there is little research in this area. I would greatly appreciate your help with this project.**

**This questionnaire should be filled out by the member of staff whom the Head has deemed to be most involved with the subject of anaphylaxis. It should take no longer than 5-10 minutes to complete.**

**Please state your position within the school:**

**Name of school**

**Region in Scotland**

**PTO**

**Please answer the following as best as possible in relation to your school:**

**PROTOCOL**

**1. Do any pupils suffer from anaphylaxis or carry an Epipen/Anapen/adrenaline auto-injector?**

Yes (please state how many)…………………................No Don't know

**If No/Don’t know, please proceed to question 3. If Yes, continue to question 2.**

| **2. If Yes, do any of these children have a personal care plan that has been developed specifically for their needs?** |  | |
| --- | --- | --- |
| Yes: Please state how many……………………………No Don't know |  |  |

| **3. Is there a standard management protocol in place in your school for emergency treatment in the event of an anaphylactic reaction?** |  | |
| --- | --- | --- |
| Yes No Don't know |  |  |

**If No/Don’t know please proceed to question 8. If Yes, continue to question 4.**

**4. Who provided the protocol?**

Local education authority
Local health board
Health professional: Please state who………………………………………………………………………………..
Other: Please state who………………………………………………………………………………………………

**5. When was the protocol introduced?**

year

**6. Has the protocol ever subsequently been reviewed?**

Yes No Don't know

**7. If yes, please state in what year/s or on what occasions:**

**IN THE EVENT OF AN EMERGENCY**

**8. Is there emergency medication on site, in case of an anaphylactic reaction?**

Yes No Don't know

**9. Who is trained to administer emergency treatment for anaphylaxis?**

School nurse All teachers

Teachers who have anaphylactic children in their classes Headteachers Class aids/support teachers No one
Some pupils: Please state for example: prefects, heads…………………………………………………………

**If no one has been trained please proceed to question 14.**

**10. How often is this training reviewed?**

Termly Yearly Other: Please state………………………………….....

**11. Who provides the training?**

School doctor School nurse Trained specialist

Other: Please state……………………………………………………………………………………………….

**12. How confident are you about the training that you and your colleagues have been given?**

Would not feel confident in an emergency situation.
Would remember the basics in an emergency situation.

Would remember most of the training in an emergency situation.

Would remember the essentials but not 100% confident in an emergency situation.

Completely confident I could handle any situation to the level of my training.

**14. Do you feel that further training on the emergency treatment of anaphylaxis would be beneficial in your school?**

Yes No Don’t Know

**If there are NO pupils in your school who suffer from food allergies/anaphylaxis, please proceed to the conclusion. If there are please continue to prevention.**

**PREVENTION
Anaphylaxis is most commonly triggered when a child is exposed to small amounts of food to which they are known to be allergic.**

**15. Is there guidance for cafeteria staff on the prevention of anaphylaxis?**

Yes No Don't know

**16. Are there any children who require elimination diets, due to their allergies?**

Yes No Don't know

**17. If yes, is their diet catered for at school?**

Yes No Don't know

**18. Is there special supervision for high risk children at eating times?**

Yes No Don't know

**19. Is there a no eating utensil sharing policy for children in place at your school?**

Yes No Don't know

**20. Have relevant lessons been reviewed, to ensure no potential trigger foods for anaphylaxis are involved?**

Yes No Don't know

**21. Is there a no eating policy on transport to and from school?**

Yes No Don't know

**22. Is there someone to enforce this?**

Yes No Don't know

**23. Is there a protocol on food provided for trips/sports days/special events?**

Yes No Don't know

**CONCLUSION**

**24. Would your school welcome further information/advice on anaphylaxis?**

Yes No Don't know

**25. Do you think that there is a need for a national set of guidelines with regard to the prevention and treatment of anaphylaxis in Scottish schools?**

Yes No Don't know

Please include any feedback that you may have, or highlight any issues that you feel need addressed relating to the care of children with anaphylaxis at school.

………………………………………………………………………………………………………………………………………………………………………………………………………………………………………………………………………………………………………………………………………………………………………………………………………………………………………………………………………………………………………………………………………………………………………………………………………………………………………………………………

**Please return to Kirsty Rankin in the SAE enclosed or fax to: 0131 650 9119**

**THANK YOU FOR YOUR TIME.**
